# Alveolar membrane and capillary function in COVID-19 convalescents: insights from chest MRI

**DOI:** 10.1007/s00330-024-10669-9

**Published:** 2024-03-09

**Authors:** Agilo Luitger Kern, Isabell Pink, Agnes Bonifacius, Till Kaireit, Milan Speth, Lea Behrendt, Filip Klimeš, Andreas Voskrebenzev, Jens M. Hohlfeld, Marius M. Hoeper, Tobias Welte, Frank Wacker, Britta Eiz-Vesper, Jens Vogel-Claussen

**Affiliations:** 1 Institute for Diagnostic and Interventional Radiology, Hannover Medical School, Carl-Neuberg-Str. 1, 30625 Hannover, Germany; 2 Biomedical Research in Endstage and Obstructive Lung Disease Hannover (BREATH), German Center for Lung Research (DZL), Carl-Neuberg-Str. 1, 30625 Hannover, Germany; 3 Medizinische Hochschule Hannover, Carl-Neuberg-Straße 1, Hannover, 30625 Germany; 4 Department of Respiratory Medicine and Infectious Diseases, Hannover Medical School, Carl-Neuberg-Str. 1, 30625 Hannover, Germany; 5 Institute of Transfusion Medicine and Transplant Engineering, Hannover Medical School, Carl-Neuberg-Str. 1, 30625 Hannover, Germany; 6 German Center for Infection Research (DZIF), Partner Site Hannover/Brunswick, Inhoffenstr. 7, 38124 Braunschweig, Germany; 7 Department of Clinical Airway Research, Fraunhofer Institute for Toxicology and Experimental Medicine, Nikolai-Fuchs-Str. 1, 30625 Hannover, Germany

**Keywords:** Thorax, COVID-19, Magnetic resonance imaging, Lung function

## Abstract

**Objectives:**

To investigate potential presence and resolution of longer-term pulmonary diffusion limitation and microvascular perfusion impairment in COVID-19 convalescents.

**Materials and methods:**

This prospective, longitudinal study was carried out between May 2020 and April 2023. COVID-19 convalescents repeatedly and age/sex-matched healthy controls once underwent MRI including hyperpolarized ^129^Xe MRI. Blood samples were obtained in COVID-19 convalescents for immunophenotyping. Ratios of ^129^Xe in red blood cells (RBC), tissue/plasma (TP), and gas phase (GP) as well as lung surface-volume ratio were quantified and correlations with CD4^+^/CD8^+^ T cell frequencies were assessed using Pearson’s correlation coefficient. Signed-rank tests were used for longitudinal and *U* tests for group comparisons.

**Results:**

Thirty-five participants were recruited. Twenty-three COVID-19 convalescents (age 52.1 ± 19.4 years, 13 men) underwent baseline MRI 12.6 ± 4.2 weeks after symptom onset. Fourteen COVID-19 convalescents underwent follow-up MRI and 12 were included for longitudinal comparison (baseline MRI at 11.5 ± 2.7 weeks and follow-up 38.0 ± 5.5 weeks). Twelve matched controls were included for comparison. In COVID-19 convalescents, RBC-TP was increased at follow-up (*p* = 0.04). Baseline RBC-TP was lower in patients treated on intensive care unit (*p* = 0.03) and in patients with severe/critical disease (*p* = 0.006). RBC-TP correlated with CD4^+^/CD8^+^ T cell frequencies (*R* = 0.61/ − 0.60) at baseline. RBC-TP was not significantly different compared to matched controls at follow-up (*p* = 0.25).

**Conclusion:**

Impaired microvascular pulmonary perfusion and alveolar membrane function persisted 12 weeks after symptom onset and resolved within 38 weeks after COVID-19 symptom onset.

**Clinical relevance statement:**

^129^Xe MRI shows improvement of microvascular pulmonary perfusion and alveolar membrane function between 11.5 ± 2.7 weeks and 38.0 ± 5.5 weeks after symptom onset in patients after COVID-19, returning to normal in subjects without significant prior disease.

**Key Points:**

• *The study aims to investigate long-term effects of COVID-19 on lung function, in particular gas uptake efficiency, and on the cardiovascular system*.

• *In COVID-19 convalescents, the ratio of *^*129*^*Xe in red blood cells/tissue plasma increased longitudinally (p* = *0.04), but was not different from matched controls at follow-up (p* = *0.25)*.

• *Microvascular pulmonary perfusion and alveolar membrane function are impaired 11.5 weeks after symptom onset in patients after COVID-19, returning to normal in subjects without significant prior disease at 38.0 weeks*.

**Supplementary Information:**

The online version contains supplementary material available at 10.1007/s00330-024-10669-9.

## Introduction

COVID-19 had and still has a great impact on the daily life of a large fraction of the world population. It is characteristic for SARS-CoV-2 that disease severity is highly variable between patients and that only mildly symptomatic or even asymptomatic patients are infectious and thus likely to spread the virus [1, 2].

Clinical imaging in COVID-19 focuses on the presence of ground-glass opacities and consolidations in the lung on CT [3, 4]. Repeated chest CTs after COVID-19 have shown residual ground-glass opacities and fibrotic changes after 6 months in a large fraction (62%) of patients with severe COVID-19 [5].

Beyond the ability to assess macroscopic structural changes [6], MRI of the lung can provide insights into functional and microstructural changes [7–10]. Li et al utilized hyperpolarized ^129^Xe MRI to study ventilation, airway morphometry, and diffusive exchange in the alveolar septa in patients after COVID-19 pneumonia [11]. An increase in ventilation defect percentage, septal gas exchange time, and reduced ratio of ^129^Xe in red blood cells (RBC) and tissue/plasma (TP) was found in comparison to healthy controls. No evidence was found for changes in airway morphometry. Similarly, later literature reports described the ratio RBC-TP to be reduced in patients after COVID-19 several months after hospital discharge compared to healthy controls [12–14]. Given the dependence of ^129^Xe gas exchange metrics on demographic variables like age [15], the interpretation of such comparisons may not always be straightforward, however. This warrants longitudinal study designs, careful matching of control groups, and assessment of prior disease with a recent literature report showing improvement of ^129^Xe gas exchange metrics between 6 and 12 weeks after hospital admission but reduction also 1 year after COVID-19 in a small group of initially 9 patients [16]. Further, Matheson et al [17] recently found a non-significant trend for increased RBC-TP between 14 and 7 months after infection in 21 patients still symptomatic at baseline.

MRI in patients recently recovered from COVID-19 revealed cardiac involvement in the majority of cases with left ventricular ejection fraction being reduced and left ventricular end-diastolic volume increased compared to healthy controls [18]. From the perspective of cellular immune response, lymphopenia has been shown to be associated with disease severity [19] and in convalescents from COVID-19 months after clinical recovery the CD4^+^ and CD8^+^ T cell compartments were found to be different compared to healthy controls [20].

We hypothesized that there would be an increase of RBC-TP ratio in patients fully recovered from COVID-19 between approximately 3 and 9 months after symptom onset. The purpose of this study was to investigate the potential presence and resolution of longer-term diffusion limitation and capillary blood volume reductions in patients after recovery from COVID-19, to correlate the findings to results from immunophenotyping, and to compare results to those from age- and sex-matched healthy controls.

## Materials and methods

This prospective study was approved by the institutional review board and written informed consent was obtained from all participants. The study population was not reported previously. The study was performed between May 2020 and April 2023.

### Participant enrollment

Two subject groups were enrolled: (1) COVID-19 convalescents and (2) age/sex-matched healthy controls. Inclusion criteria for (1) were previous COVID-19 infection as determined by positive swab test for SARS-CoV-2. Exclusion criteria were missing written informed consent, known history of chronic obstructive lung disease as well as interstitial lung disease, and known immunodeficiency (for longitudinal analysis). All patients hospitalized between May 2020 and November 2020 were offered clinical follow-up at the post-COVID outpatient clinic in the Department of Pneumology at Hannover Medical School. In addition, the patients were offered a lung MRI as part of this study. For group (2), healthy subjects with less than 1 pack-year of smoking history were recruited. Exclusion criteria were current lung infection and history of lung surgery. Exclusion criteria for both groups were pregnancy or breast-feeding, and general MRI contraindications (metallic implant/claustrophobia).

### Study design

COVID-19 convalescents were recruited after recovery from COVID-19 and underwent lung MRI with collection of blood samples for immunophenotyping around 3 and 9 months after symptom onset. Lung function tests including body plethysmography (performed according to European Respiratory Society standards [21]), blood gas analysis (blood obtained at rest from one earlobe), symptom assessment, and CT were performed as part of the clinical follow-up. Two participants were additionally examined at hospital discharge. Further, healthy controls age/sex-matched to the COVID-19 convalescents included at follow-up were recruited and underwent ^129^Xe MRI.

### Patient groups

COVID-19 convalescents were divided into groups treated on intensive care unit (ICU) versus those not treated on ICU or in an ambulatory setting. In an additional analysis, the patients’ disease severity was classified according to World Health Organization guidelines [22], dividing patients into groups with mild/moderate and severe/critical disease.

### Immunophenotyping

Details of the methods for immunophenotyping are described in the supplemental material.

### Imaging methods

MR imaging was performed at 1.5 T (Magnetom Avanto, Siemens Healthcare GmbH). For ^1^H dynamic contrast-enhanced and cardiac imaging, torso (6-channel) and spine (24-channel) coils were used. For ^129^Xe imaging, a custom-made linearly polarized birdcage coil (transmission) and a 16-channel phased-array (reception) were used (Rapid Biomedical GmbH). ^129^Xe was hyperpolarized (Polarean 9810, Polarean Imaging plc.) and dispensed into Tedlar bags (Jensen Inert Products).

The first ^129^Xe bag was used for low-resolution ventilation imaging and subsequent transmitter calibration. Hyperpolarized ^129^Xe dissolved phase imaging and dynamic dissolved phase spectroscopy were performed using a second bag as described in the supplemental material. Using a third bag, diffusion-weighted imaging at short diffusion times [10] and chemical shift saturation recovery (CSSR) spectroscopy were performed. Hyperpolarized ^129^Xe dissolved phase imaging was performed using a 3D-radial multi-gradient echo sequence with TR/TE 18 ms/(0.61/1.42/2.23/3.04/3.85 ms), flip angle dissolved/gas 21°/0.4°, and bandwidth 650 Hz/px. Dynamic spectroscopy of the dissolved phase was performed with frequency selective excitation near the RBC resonance at 222.5 ppm, TR/TE 36 ms/1.2 ms, spectral resolution 32.5 Hz, 160 measurements, and flip angle 60°. Chemical shift saturation recovery (CSSR) delay times ranged from 3 to 600 ms. Dissolved phase magnetization was destroyed using a pair of 2.4-ms-long rectangular RF pulses centered on the TP and RBC resonances at 198 and 218 ppm, respectively, with 200-µs gap. Transmit voltages were increased relative to a nominal 90° flip angle by an empirical correction factor of ~ 1.2 in order to avoid a vertical offset in CSSR uptake curves. Diffusion-weighted imaging included diffusion times 920, 1140, 1400, 1660, and 1960 µs at a constant *b*-value of 3 s/cm^2^. Both diffusion-weighted imaging and CSSR were performed at full lung inflation.

Cardiac MRI and dynamic contrast-enhanced MRI methods are described in the supplemental material. Clinical chest CT was used for comparison to MRI if a current CT (within 1 month of the MRI) was available.

### Data analysis

The ratios of peak integrals RBC-TP, RBC-GP, and TP-Gas in dynamic spectroscopy were formed and the function

1 $$f\left(\overrightarrow{p},n\right)={p}_{1}\left(1+{p}_{2}{\text{sin}}\left({p}_{3}n+{p}_{4}\right)\right)+{p}_{5}\left(n-\frac{{N}_{{\text{meas}}}}{2}\right)$$

was fit to the data with *n* the number of the measurement and *N*_meas_ the total number of measurements.

In diffusion-weighted imaging, whole-lung averages for the ^129^Xe apparent diffusion coefficient *D*_app_ were computed and the lung surface-volume ratio S_a_/V_g_ was quantified [10, 23].

For CSSR analysis, a generalized CSSR model [24]

2 $$F\left(t\right)=\frac{\lambda L}{2}\eta \frac{{\text{S}}}{V}\frac{\tau -t}{\tau }\left[1-\sum\limits_{n=1}^{\infty }\frac{{\left(-1\right)}^{n+1}+1}{{B}^{2}+{\mu }_{n}^{2}+2B}\frac{4{B}^{2}}{{\mu }_{n}^{2}}{e}^{\frac{-D{\mu }_{n}^{2}}{{L}^{2}}t}\right]+\lambda L\eta \frac{S}{V}\frac{1}{\tau }\left[t+\sum\limits_{n=1}^{\infty }\frac{{\left(-1\right)}^{n+1}+1}{{B}^{2}+{\mu }_{n}^{2}+2B}\frac{4{B}^{2}{L}^{2}}{D{\mu }_{n}^{4}}\left({e}^{\frac{-D{\mu }_{n}^{2}}{{L}^{2}}t}-1\right)\right]$$

fit to the data in order to extract the diffusional Biot number *B* describing membrane permeability, RBC fraction *η*, and the capillary transit time *τ*.

Analysis of cardiac MRI and dynamic contrast-enhanced MRI and further details of ^129^Xe MRI analysis are described in the supplemental material. CT images were automatically analyzed for lung disease patterns as well as mean CT value using AVIEW (Coreline Soft, Co., Ltd.). The fraction of lung parenchyma with ground-glass opacities not including consolidation and reticulation within the whole lung was calculated.

### Statistical analysis

Data are shown as mean ± standard deviation if not noted otherwise. Statistical testing for significance of longitudinal changes was performed using Wilcoxon signed-rank tests. For group comparison, Mann–Whitney *U* tests were used. Correlations were assessed using Pearson’s correlation coefficient and significance of correlation tested by permutation tests. Statistical analysis was performed by AK using Matlab R2021a (The MathWorks, Inc.). The significance level was set at *p* < 0.05 two-sided.

## Results

### Study population

**Fig. 1:**
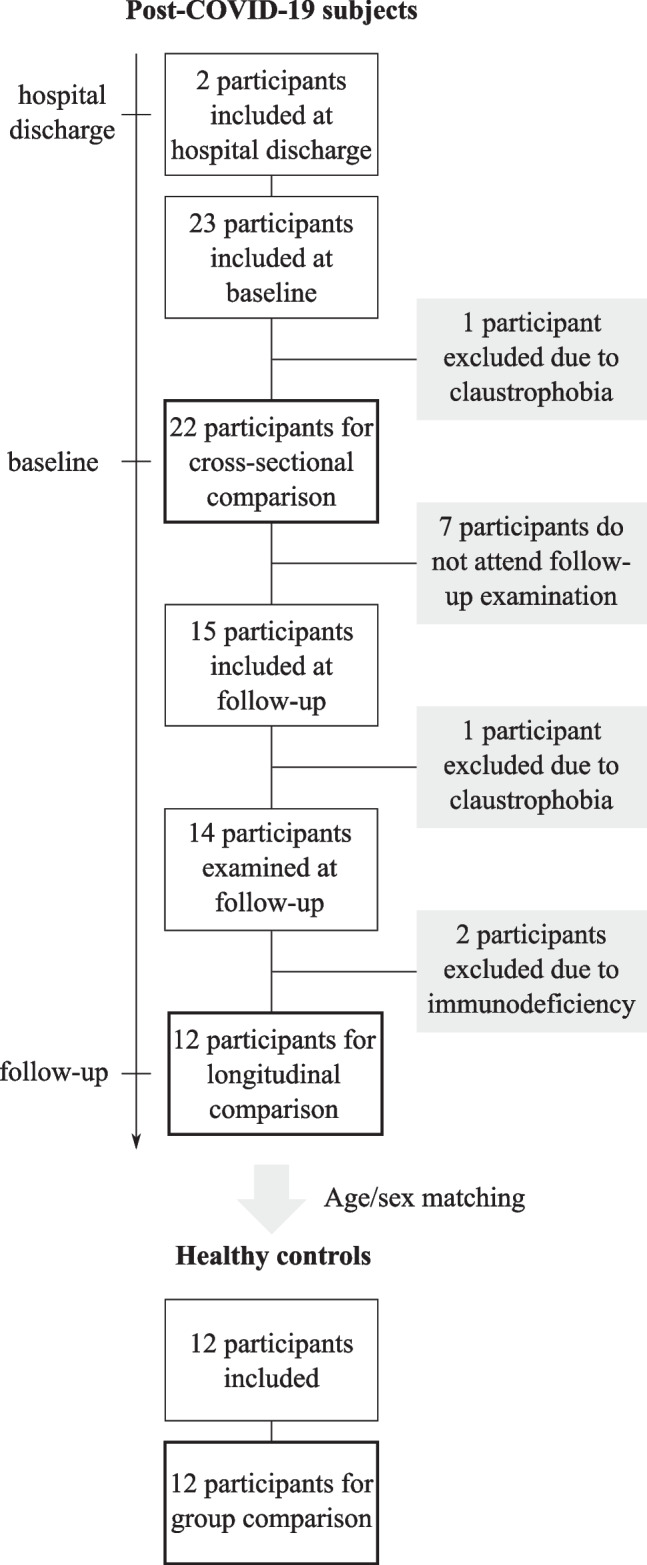
Study flow diagram. Flow diagram describing inclusion and exclusion of participants for both post-COVID-19 subjects and healthy controls. Time points after symptom onset were 4.5 ± 1.1 weeks for hospital discharge, 12.6 ± 4.2 weeks for baseline examination, and 38.0 ± 5.5 weeks for the follow-up examination

A total of 35 participants for the two groups of COVID-19 convalescents and matched healthy controls (mean age at inclusion, 51.2 ± 15.7 years, 20 men) were included. At baseline, one participant in the COVID-19 convalescents group, initially 23 subjects, suffered from claustrophobia and was excluded from the study. Seven subjects did not attend the follow-up examination and in 2 of these subjects ^129^Xe MRI could not be performed at baseline due to technical issues. For the 15 remaining subjects, a current clinical chest CT was available at baseline. Fifteen participants in the COVID-19 convalescents group participated in the follow-up examination out of which one suffered from claustrophobia and was excluded. Two participants were excluded from longitudinal analysis due to history of immunodeficiency. A flow diagram describing participant enrollment/exclusion and time of MRI is shown in Fig. 1. Subject demographics and clinical characteristics for the COVID-19 convalescents are summarized in Table 1, and for the longitudinally compared COVID-19 convalescents and matched controls in Table 2. Results from symptom assessment in COVID-19 convalescents are reported in Table 3. In brief, results from lung function tests in 22 participants at baseline indicate mild reduction of FVC and D_LCO_; however, more than half of the subjects still reported symptoms.

**Table 1:** Post-COVID-19 subject^a^ demographics, clinical characteristics, and comorbidities at baseline

Quantity	Value (all patients)
Sex (male/female)	13/9
Age (years)	52.9 ± 19.5
FEV_1_ (% of predicted)	93.8 ± 14.5
FVC (% of predicted)	91.3 ± 18.9
FEV_1_/FVC (%)	78.5 ± 5.4
D_LCO_ (% of predicted)	85.1 ± 23.7
K_CO_ (% of predicted)	97.2 ± 19.7
Number hospitalized patients	16
Length of hospitalization^b^ (days)	16.4 ± 12.9
Time after hospital discharge^b^ (weeks)	8.4 ± 3.2
Time after symptom onset (weeks)	12.6 ± 4.2
CT: time after symptom onset (weeks)	11.7 ± 4.2
Smoking history (pack-years)	4.0 ± 7.7
Comorbidity category	Number of patients with comorbidity (fraction in %)
Adiposity	3 (14%)
Cardiovascular	2 (9%)
Diabetes	2 (9%)
Depression	1 (5%)
Hypertension	6 (27%)
Liver disease	1 (5%)
Renal insufficiency	0 (0%)
Immunodeficiency	4 (18%)

^a^22 participants, ^b^if at hospital. *D*_*LCO*_ diffusing capacity of the lung for carbon monoxide, *FEV*_*1*_ forced expiratory pressure in 1 s, *FVC* forced vital capacity, *K*_*CO*_ carbon monoxide transfer coefficient

**Table 2:** Longitudinal comparison of clinical characteristics in post-COVID-19 subjects^a^ as well as group comparison at follow-up to matched healthy controls^b^

	Post-COVID-19 subjects			Matched healthy controls	
Quantity	Value at baseline	Value at follow-up	*p* value	Value	*p* value
Age (years)	49.1 ± 15.6	49.6 ± 15.7	n/a	49.4 ± 14.4	0.93
Body mass index (kg/m^2^)	25.3 ± 3.8	26.3 ± 4.0	0.16	25.1 ± 2.7	0.53
FEV_1_ (% of predicted)	97.0 ± 11.8	95.1 ± 12.3	0.26	95.3 ± 7.9	1.00
FVC (% of predicted)	99.5 ± 12.3	101.9 ± 13.2	**0.03**	103.8 ± 8.5	0.60
FEV_1_/FVC (%)	77.8 ± 5.2	74.4 ± 6.2	**0.02**	75.8 ± 5.2	0.67
D_LCO_ (% of predicted)	94.8 ± 23.2	94.0 ± 15.0	0.79	–	n/a
K_CO_ (% of predicted)	103.1 ± 20.3	102.4 ± 13.4	0.89	–	n/a
Time after hospital discharge^c^ (weeks)	7.5 ± 2.2	34.5 ± 5.1	n/a	n/a	n/a
Time after symptom onset (weeks)	11.5 ± 2.7	38.0 ± 5.5	n/a	n/a	n/a

^a^12 participants, 7 male, 6 hospitalized, 2 treated on intensive care unit, ^b^12 participants, 7 male, ^c^if at hospital. Statistically significant *p* values are indicated in bold. *D*_*LCO*_ diffusing capacity of the lung for carbon monoxide, *FEV*_*1*_ forced expiratory pressure in 1 s, *FVC* forced vital capacity, *K*_*CO*_ carbon monoxide transfer coefficient

**Table 3:** Assessment of symptoms in post-COVID-19 subjects

	All subjects (*n* = 22)	Subjects for longitudinal comparison (*n* = 12)	
Symptom category	Number of subjects reporting symptom (fraction in %)		
	Baseline	Baseline	Follow-up
Angina	7 (32%)	5 (42%)	4 (33%)
Arthritis	2 (9%)	1 (8%)	0 (0%)
Cough	5 (23%)	4 (33%)	2 (17%)
Fever	0 (0%)	0 (0%)	0 (0%)
Headache	7 (32%)	4 (33%)	3 (25%)
Hemoptysis	0 (0%)	0 (0%)	0 (0%)
Night sweats	6 (27%)	2 (17%)	1 (8%)
Reflux	4 (18%)	3 (25%)	2 (17%)
Sickness	1 (5%)	0 (0%)	2 (17%)
Sputum	3 (14%)	1 (8%)	1 (8%)
Syncope	0 (0%)	0 (0%)	0 (0%)
Weight loss	3 (14%)	1 (8%)	0 (0%)
Any	14 (64%)	9 (75%)	5 (42%)

### Longitudinal comparison

**Fig. 2:**
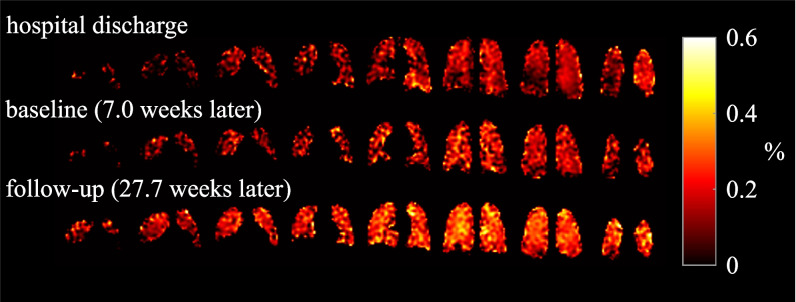
Exemplary imaging results from ^129^Xe dissolved phase imaging in longitudinal comparison. RBC-GP ratio maps from dissolved phase imaging in a 32-year-old male patient in longitudinal comparison showing increasing RBC-GP with increasing time after hospital discharge mentioned in brackets. Dates correspond to 3.7 weeks, 10.7 weeks, and 31.4 weeks after symptom onset. Abbreviations: GP, gas phase; RBC, red blood cell

Figure 2 shows imaging results from dissolved phase imaging in a participant who received an additional MRI at hospital discharge.

The average RBC-GP ratio in dynamic spectroscopy significantly increased from baseline to follow-up (0.145 ± 0.038 vs. 0.157 ± 0.037, *p* = 0.01). Similarly, the average RBC-TP ratio equivalent to *p*_1_ in Eq. 1 increased (0.925 ± 0.196 to 0.999 ± 0.139, *p* = 0.04) as well as absolute oscillation amplitude *p*_1_*p*_2_ (*p* = 0.03), whereas relative oscillation amplitude *p*_2_ did not (*p* = 0.97). No longitudinal change was observed in the TP-Gas ratio (*p* = 0.95). The average full width at half maximum of the RBC line decreased from baseline to follow-up (212 ± 10 Hz vs. 207 ± 7 Hz, *p* = 0.02).

**Fig. 3:**
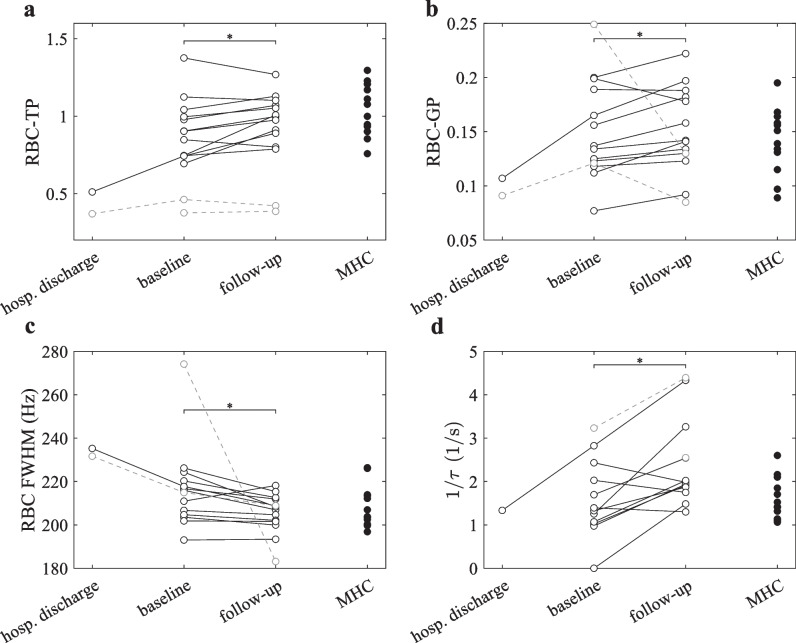
Longitudinal comparison in post-COVID-19 subjects and group comparison with matched healthy controls with respect to parameters from ^129^Xe dissolved phase. Longitudinal changes in (**a**) RBC-TP ratio, (**b**) RBC-GP ratio, (**c**) RBC FWHM from dynamic spectroscopy, and (**d**) 1/τ from CSSR spectroscopy in post-COVID-19 subjects (black circles) as well as comparison to corresponding values in matched healthy controls (black dots). Gray circles and dashed lines denote data from participants excluded from longitudinal analysis due to prior disease of the immune system. Baseline is 7.5 ± 2.2 weeks, follow-up 34.5 ± 5.1 after hospital discharge in hospitalized patients. Abbreviations: CSSR, chemical shift saturation recovery; FWHM, full width at half maximum; GP, gas phase; MHC, matched healthy controls; RBC, red blood cell; TP, tissue/plasma; τ, capillary transit time

Lung surface-volume ratio from diffusion-weighted imaging did not significantly change in longitudinal comparison (*p* = 0.38). In CSSR spectroscopy, the inverse capillary transit time 1/*τ* proportional to RBC velocity increased between baseline and follow-up (1.46 ± 0.77 1/s vs. 2.22 ± 0.88 1/s, *p* = 0.01). No significant change was observed for the Biot number *B*/membrane permeability or RBC fraction *η* (*p* = 0.76 and 0.21, respectively). Figure 3 summarizes findings from dynamic spectroscopy and CSSR spectroscopy.

No significant longitudinal changes were observed in ventilation defect percentage from ^129^Xe ventilation imaging (*p* = 0.21). In dissolved phase imaging, an increase of RBC-GP ratio between baseline and follow-up (0.239 ± 0.094% vs. 0.271 ± 0.074%, *p* = 0.02) was observed in the study population. The change in RBC-TP ratio was not significant, however (*p* = 0.12).

Analysis of pulmonary microvascular perfusion from dynamic contrast-enhanced imaging and heart function from cardiac MRI showed no significant changes. The supplemental material contains a table summarizing longitudinal changes of MRI parameters.

No significant longitudinal changes in lymphocyte counts were observed. Lung function tests showed a significant increase of forced vital capacity (FVC) as percent of predicted value (*p* = 0.03), but no significant change in forced expiratory volume in 1 s (FEV_1_) as percent of predicted (*p* = 0.26). The FEV_1_/FVC ratio was significantly lower at follow-up (*p* = 0.02). No significant change was observed in diffusing capacity for carbon monoxide (D_LCO_) as percent of predicted (*p* = 0.79) and for the carbon monoxide transfer coefficient K_CO_ as percent of predicted (*p* = 0.89).

### Comparison with matched healthy controls at follow-up

**Fig. 4:**
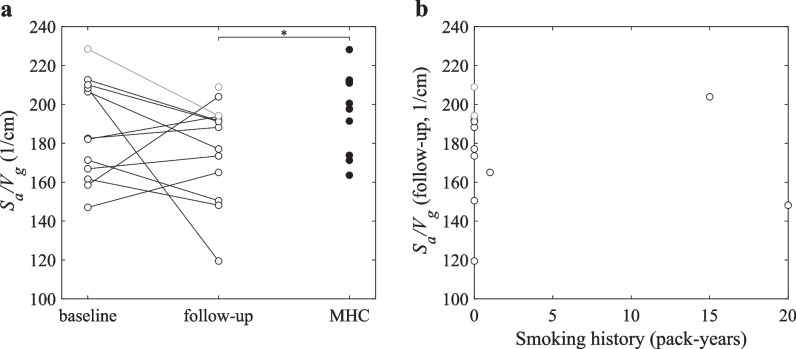
Results from surface-volume ratio measurements from ^129^Xe diffusion-weighted imaging. **a** Longitudinal and group comparison of lung surface-volume ratio S_a_/V_g_ in COVID-19 convalescents (black circles) at baseline and follow-up and matched healthy controls (black dots). Gray circles and lines denote data from participants excluded from longitudinal analysis due to prior disease of the immune system. There was no significant longitudinal change of surface-volume ratio (*p* = 0.38); however, surface-volume ratio was significantly reduced compared to matched healthy controls (*p* = 0.02). **b** There was no significant correlation between surface-volume ratio at follow-up and smoking history (*R* =  − 0.02, *p* = 0.95). Abbreviations: MHC, matched healthy controls; S_a_/V_g_, lung surface-volume ratio

Lung surface-volume ratio from ^129^Xe diffusion-weighted imaging was significantly reduced in COVID-19 convalescents compared to matched healthy controls (173 cm^−1^ ± 25 cm^−1^ vs. 199 cm^−1^ ± 20 cm^−1^, *p* = 0.02). There was a non-significant trend for increased inverse capillary transit time in COVID-19 convalescents (*p* = 0.06). No other significant differences in functional MRI metrics were observed. Although there were no active smokers, COVID-19 convalescents in this group had a smoking history of 3.4 pack-years ± 6.8 pack-years compared to never-smoking healthy controls. There was no correlation between smoking history and surface-volume ratio from diffusion-weighted imaging at follow-up, however (*R* =  − 0.02, *p* = 0.95) (see Fig. 4).

### Cross-sectional comparison

**Fig. 5:**
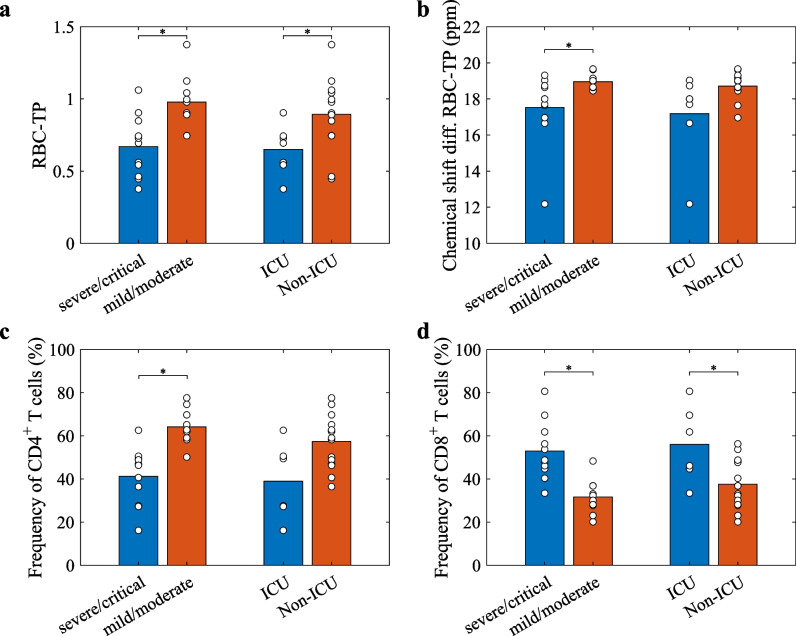
Cross-sectional comparisons between post-COVID-19 patient subgroups at baseline. Group comparison at baseline visit between patients with severe/critical and mild/moderate disease as well as between patients treated on ICU and others for (**a**) RBC-TP from dynamic spectroscopy, (**b**) chemical shift difference between RBC-TP, (**c**) frequency of CD4^+^ T cells, and (**d**) CD8^+^ T cells. Abbreviations: CD, cluster of differentiation; ICU, intensive care unit; ppm, parts per million; RBC, red blood cell; TP, tissue/plasma

Results of cross-sectional comparison are summarized in Fig. 5. At baseline, the average RBC-TP ratio from dynamic spectroscopy was reduced in patients after intensive care treatment compared to those not requiring intensive care (0.650 ± 0.171 vs. 0.894 ± 0.256, *p* = 0.03). Similarly, patients with WHO disease severity classification severe/critical had reduced RBC-TP compared to those with WHO disease severity classification mild or moderate (0.670 ± 0.214 vs. 0.978 ± 0.195, *p* = 0.006). The difference in chemical shift between RBC and TP was not significant comparing patients treated on ICU and others (*p* = 0.07), but it was significant in the comparison of patients with severe/critical and moderate/mild disease (*p* = 0.03). The relative oscillation amplitude *p*_1_ of RBC-TP was significantly increased in patients with severe/critical disease (*p* = 0.03), but not in patients treated on ICU compared to others (*p* = 0.87). No significant difference of ventilation defect percentage was observed between ICU and non-ICU patients (*p* = 0.58), as well as patients with severe/critical and mild/moderate disease (*p* = 0.44).

The frequency of CD4^+^ T cells tended to be reduced in ICU patients and was significantly reduced in patients with severe/critical disease (*p* = 0.07 and *p* < 0.001, respectively). On the contrary, CD8^+^ T cell frequency was increased (*p* = 0.04 and *p* < 0.001). FVC as percent of predicted value was reduced in patients treated on ICU (*p* = 0.02) and with severe/critical disease (*p* < 0.001). Clinical characteristics in cross-sectional comparison are summarized in Table 4.

**Table 4:** Cross-sectional subgroup comparison of clinical characteristics at baseline

Quantity	Patients on intensive care unit	Patients not on intensive care unit	*p* value	Patients with severe/critical disease	Patients with mild/moderate disease	*p* value
Number (male/female)	7 (6/1)	15 (7/8)	n/a	12 (9/3)	10 (4/6)	n/a
Number hospitalized patients	7	9	n/a	12	4	n/a
Age (years)	49.3 ± 11.6	54.5 ± 18.5	0.53	57.8 ± 15.6	46.9 ± 16.3	0.11
FEV_1_ (% of predicted)	85.8 ± 16.0	97.5 ± 12.6	0.19	89.2 ± 16.5	100.3 ± 8.3	0.09
FVC (% of predicted)	76.9 ± 22.8	99.1 ± 10.9	**0.02**	82.0 ± 18.5	105.3 ± 7.7	** < 0.001**
FEV_1_/FVC (%)	81.0 ± 4.5	77.3 ± 5.5	0.07	80.0 ± 6.5	76.4 ± 2.3	0.19
D_LCO_ (% of predicted)	64.6 ± 13.0	92.9 ± 22.3	**0.01**	75.6 ± 22.9	96.9 ± 20.1	**0.046**
K_CO_ (% of predicted)	81.8 ± 9.1	103.1 ± 19.6	**0.04**	94.3 ± 19.3	100.8 ± 20.9	0.53
Length of hospitalization^a^ (days)	24.4 ± 13.7	6.1 ± 8.2	0.13	20.8 ± 12.0	1.4 ± 2.1	** < 0.001**
Time after hospital discharge^b^ (weeks)	8.1 ± 2.5	8.6 ± 3.6	0.17	9.1 ± 3.4	7.0 ± 2.1	0.36
Time after symptom onset (weeks)	11.9 ± 2.5	12.7 ± 4.8	0.89	12.7 ± 3.5	12.1 ± 5.0	0.41

^a^0 if not hospitalized, ^b^if at hospital. Statistically significant *p* values are indicated in bold. *D*_*LCO*_ diffusing capacity of the lung for carbon monoxide, *FEV*_*1*_ forced expiratory pressure in 1 s, *FVC* forced vital capacity, *K*_*CO*_ carbon monoxide transfer coefficient

### Correlations at baseline

**Fig. 6:**
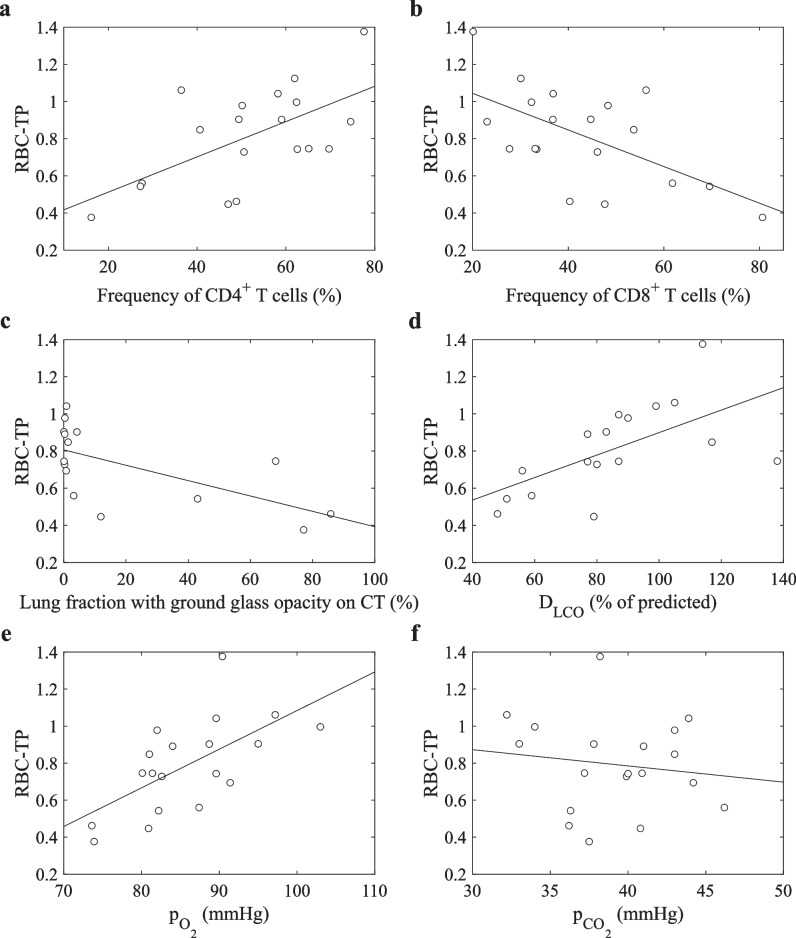
Correlations of ^129^Xe MRI with immunophenotyping and clinical parameters. Correlation of data at baseline visit and linear regressions: Average RBC-TP ratio from dynamic spectroscopy is significantly correlated with (**a**) frequency of CD4^+^ T cells (*R* = 0.61, *p* = 0.006), (**b**) frequency of CD8^+^ T cells (*R* =  − 0.60, *p* = 0.006), (**c**) fraction of lung volume with ground-glass opacity on CT (*R* =  − 0.63, *p* = 0.01), (**d**) D_LCO_ as percent of predicted value (*R* = 0.61, *p* = 0.01) as well as (**e**) p_O2_ from blood gas analysis (*R* = 0.63, *p* = 0.003). No significant correlation was found in the case of (**f**) p_CO2_ (*R* =  − 0.14, *p* = 0.58). Abbreviations: CD, cluster of differentiation; D_LCO_, diffusing capacity of the lung for carbon monoxide; RBC, red blood cell; TP, tissue/plasma

At baseline, RBC-TP ratio from dynamic spectroscopy was positively correlated with the frequency of CD4^+^ T cells (*R* = 0.61, *p* = 0.006) and negatively correlated with the frequency of CD8^+^ T cells (*R* =  − 0.60, *p* = 0.006). Similarly, RBC-TP ratio was negatively correlated with the fraction of the lung volume determined as ground-glass opacity by texture analysis on CT (*R* =  − 0.63, *p* = 0.01). Average time between MRI and CT examination is 0.0 days ± 14.1 days. Significant correlations between RBC-TP ratio and D_LCO_ as percent of predicted value (*R* = 0.61, *p* = 0.01), arterial oxygen partial pressure from blood gas analysis (*R* = 0.63, *p* = 0.003), but not carbon dioxide partial pressure (*R* =  − 0.14, *p* = 0.58) were observed (see Fig. 6). The correlation between MRI-derived surface-volume ratio and mean CT value was weak and non-significant (*R* = 0.28, *p* = 0.43).

## Discussion

The main purpose of this study was to verify the possible presence of diffusion limitation and microvascular perfusion impairment at several weeks after recovery and their resolution at later times in longitudinal comparison and comparison to matched healthy controls. We found significant differences in red blood cell to tissue/plasma ratio and red blood cell to gas-phase ratio from dynamic ^129^Xe spectroscopy when comparing 11.5 and 38.0 week after symptom onset results.

The initially reduced RBC-TP and RBC-GP ratios are likely attributable to impaired perfusion of the lung capillaries due to endothelialitis, thrombosis, and angiogenesis [25] and possibly also in part due to an increased air-blood barrier, whose effects diminish over time. The fact that no significant longitudinal changes were observed in heart function suggests that the observed difference in capillary transit time is likely due to local restrictions of capillary perfusion or inflammatory vasodilation in the lung. The comparison to matched healthy controls suggests that these values return back to normal in subjects without significant prior disease although it is very interesting to note that in the two subjects with pre-existing immunodeficiency in the group of COVID-19 convalescents RBC-TP still appears greatly reduced at follow-up as seen in Fig. 3. There may be patient groups with very slow recovery from COVID-19 as also suggested by the data reported by Matheson et al [17] in patients still symptomatic 7 months after infection.

The result of the reduction of the linewidth of the RBC resonance in longitudinal comparison is a bit surprising given that faster chemical exchange would lead to line broadening and Grist et al observed a reduced linewidth in patients compared to healthy controls [12]. The narrowing is, however, consistent with the observation of longitudinally increased blood flow velocity assuming an influence of pseudo-diffusion effects on the observed lineshapes [26]. Another possible explanation could be that the RBC resonance actually consists of multiple shifted and overlapping resonances with varying intensity, e.g., due to varying blood oxygenation which may improve over time [27].

Contrary to previous reports employing different methods of ^129^Xe diffusion-weighted imaging [11, 16], our results indicate a reduced lung surface-volume ratio in patients after COVID-19 compared to matched healthy controls about 9 months after symptom onset. Combined with the fact that lung surface-volume ratio did not significantly change longitudinally, this could suggest that there may have been damage to the lung microstructure already before COVID-19. A reduced lung surface-volume ratio could be a sign of early emphysema as it may be associated, e.g., with smoking. On the other hand, the smoking history in the post-COVID-19 group seems minor on average and in principle COVID-19 could have led to changes of surface-volume ratio between the time of disease onset and baseline MRI.

The fact that no longitudinal changes were observed for metrics of carbon monoxide diffusion is likely due to the variability of the test and relatively small sample size since in larger patient cohorts such changes were reported [28]. Further, the metrics derived from ^129^Xe MRI more specifically probe gas uptake and perfusion at the level of alveolar septa and capillaries. This may also explain why no changes in lung perfusion were observed in dynamic contrast-enhanced MRI.

The difference in RBC-TP ratio between study subgroups can be understood by assuming more frequent formation of microthrombi and thus greater impairment of capillary perfusion with increasing disease severity. The additionally observed reduced chemical shift difference between RBC and TP in more severely affected patients could be due to reduced blood oxygenation within the lung [27].

The frequencies of CD4^+^ and CD8^+^ T cells were found to be different between patients with different disease severity whereas no evidence for longitudinal change was observed. Breton et al [20] described a reduced frequency of CD4^+^ T cells and an increased frequency of CD8^+^ T cells in patients at 1.3 months after COVID-19 compared to healthy controls and compared to 6.1 months after COVID-19 in a larger patient cohort. The observed correlations between RBC-TP ratio and CD4^+^ as well as CD8^+^ T cell frequencies suggest that altered immunological signatures may have an influence on capillary function in COVID-19. Specifically, a reduction of CD4^+^ T cell count is considered as an indicator of immunosuppression and was previously found at time of hospital admission to be associated with necessity of ICU treatment [29].

As the COVID-19 patients included in this study were infected in 2020 where there appeared to be relatively little evolution of SARS-CoV-2, results of this study may not be generalizable to strains arising later in the pandemic, which may be associated with reduced disease severity [30].

The small sample size, partly caused by the comparatively large fraction of subjects not returning to the follow-up visit, should be considered a limitation of the present study. A general limitation of a study including participants after recovery from disease for investigating disease effects is that pre-COVID lung function cannot be assessed. Consequently, it is difficult to determine whether the disease has led to permanent damage. In addition, healthy subjects were matched only for age and sex, and thus the impact of comorbidities like hypertension and cardiovascular disease is not known although the return of RBC-TP to normal is not suggestive of long-term effects on the vasculature and thus influence could be restricted to increased susceptibility for the disease.

## Conclusions

We found evidence for an improvement of pulmonary capillary perfusion and alveolar gas uptake in patients recovering from COVID-19 between 11.5 ± 2.7 and 38.0 ± 5.5 weeks after symptom onset. Alveolar membrane function returns to normal values in subjects without significant prior disease although a notable fraction of subjects still reports symptoms, suggesting that these symptoms may not be a consequence of pulmonary changes. We also found evidence for a mildly reduced (13% from healthy controls) lung surface-volume ratio in COVID-19 convalescents, which might be associated with pre-COVID changes.

## Supplementary Information

Below is the link to the electronic supplementary material.

Supplementary file1 (PDF 307 KB)

## Abbreviations

CSSR: Chemical shift saturation recovery
D_LCO_: Diffusing capacity of the lung for carbon monoxide
FEV_1_: Forced expiratory volume in 1 s
FVC: Forced vital capacity
FWHM: Full width at half maximum
GP: Gas phase
ICU: Intensive care unit
K_CO_: Carbon monoxide transfer coefficient
RBC: Red blood cell
TP: Tissue/plasma

## Declarations

### Guarantor

The scientific guarantor of this publication is Agilo Luitger Kern.

### Statistics and biometry

No complex statistical methods were necessary for this paper.

### Study subjects or cohorts overlap

No study subjects or cohort overlap has been reported.

### Methodology

• prospective

• observational

• performed at one institution
