# Supplementary material for: Alveolar membrane and capillary function in COVID-19 convalescents: insights from chest MRI
Source: Eur Radiol. 2024 Mar 9;34(10):6502–13. doi: 10.1007/s00330-024-10669-9 (PMC11399308; doi:10.1007/s00330-024-10669-9)
Supplement: Supplementary file 1 — Supplementary file1 (PDF 307 KB) [file 330_2024_10669_MOESM1_ESM.pdf]

# Alveolar membrane and capillary function in COVID-19 convalescents: Insights from chest MRI – Supplemental Material

## Materials and Methods

*Immunophenotyping*

For immunophenotyping, EDTA blood was stained with anti-CD45 allophycocyanin-H7 (APC-H7), anti-CD3 fluorescein isothiocyanate (FITC), anti-CD8 allophycocyanin (APC), anti-CD4 peridinin chlorophyll protein complex (PerCP), anti-CD19 AlexaFluor 700, anti-CD56 phycoerythrin (PE), anti-γδTCR PE/Cyanine7 and anti-CD14 Brilliant Violet (BV510) (BD Biosciences, Heidelberg, Germany), followed by red blood cell lysis using 1x Lysing Solution (BD Biosciences). Samples were acquired on a 10 color BD FACSCanto flow cytometer (BD Biosciences). Data were analyzed using BD FACSDiva software version 8.0.1 (BD Biosciences).

*Imaging Methods*

Regional lung perfusion was evaluated by three-dimensional dynamic contrast-enhanced (DCE) time-resolved angiography with stochastic trajectories volume-interpolated breath-hold examination (TWIST-VIBE) using the following MR imaging parameters: TR/TE 2.85/0.72 ms; flip angle 20°; 50 three-dimensional data sets with an update rate of 1.0–1.2 s; acquisition matrix 224 × 182; field of view 50 cm × 40.6 cm; 0.04 mmol/kg gadoteric acid at 5cc/s i.v.; 44–52 reconstructed coronal slices (slice thickness 5 mm) covering the whole lung were acquired in a single breath-hold.

Cardiac function was evaluated by retrospectively ECG-gated cine balanced steady-state free precession sequences during short inspiratory breath holds using short-axis views covering the whole heart with following MRI parameters: TR/TE 43.35/1.22 ms; flip angle 77; slice thickness 8 mm; field of view 29.2 cm × 36.0 cm; matrix size 208 × 256; temporal resolution 35 ms; bandwidth/pixel 930 Hz/pixel; 30 reconstructed phases.

*Data Analysis*

$^{129}$Xe dissolved-phase imaging data were reconstructed using the parallel imaging/compressed sensing routine of Berkeley Advanced Reconstruction Toolbox (1). Reconstructed images of the $^{129}$Xe dissolved phase were then separated in RBC and TP signals using hierarchical IDEAL (2). Masks were generated based on an SNR threshold and voxel values summed within the mask to obtain whole lung values for dissolved-phase ratios.

Data from dynamic spectroscopy were apodized by a von Hann window, zero-filled to two-fold resolution, Fourier-transformed and the first 15 spectra discarded. Zeroth order phase correction was performed by applying DISPA to the gas-phase resonance (3). Real parts of complex Lorentzians were fit to the real parts of the spectra for RBC, TP and gas resonance.

In diffusion-weighted imaging, a function of the form

$$D_{\mathrm{app}}(\Delta) = D_0\left(1 - \alpha \frac{S_{\mathrm{a}}}{V_{\mathrm{g}}}\sqrt{D_0 \Delta} + B' D_0 \Delta\right) \quad \text{Eq. A1}$$

corresponding to the second-order approximation in the analysis of time-dependent diffusion by Mitra et al. was fit to the data in order to determine lung surface-volume ratio $S_a/V_g$. Here, Δ denotes the diffusion time, $D_0 = 0.14$ cm$^2$/s denotes the free diffusion coefficient of $^{129}$Xe dilute in air, $\alpha = 0.289$ denotes a constant depending on diffusion-weighting gradient lobes, and B’ an additional fitting parameter depending on lung microstructure but not readily interpretable in terms of histologic quantities.

CSSR data were Fourier-transformed and phase-corrected to zeroth and first order and peak amplitudes in real parts of the spectra integrated numerically. The ratio of RBC and GP amplitudes was computed and the model function F fit to the data.

First-pass DCE MR images were used for calculating parenchymal microvascular pulmonary blood flow maps by using a pixel-by-pixel deconvolution analysis with a home-written plugin for Visage 7 software (Visage Imaging, Inc.). Arterial input function was derived with a region of interest in the main pulmonary artery. Regions of interest were drawn on the lung perfusion maps excluding larger pulmonary vessels. Mean parenchymal pulmonary blood flow was calculated for the total lung.

Short-axis cine MR images were analyzed with dedicated software (CVI42 software, Circle Cardiovascular Imaging Inc.). Cine images were analyzed by semiautomated contour detection for left-ventricular and right-ventricular endo- and epicardial contours in end-diastole and end-systole by TK. Papillary muscles as well as myocardial trabeculations were included in the blood pool (4). Manual corrections were performed if necessary. Using short-axis cine images, the following parameters were evaluated for the left ventricle and the right ventricle: stroke volume, ejection fraction, end-diastolic volume and end-systolic volume, myocardial mass. Volumes and masses were normalized to body surface area.

## Results

*Supporting Table. Longitudinal comparison of MRI parameters in post-COVID-19 subjects (12 participants, 7 male, 6 hospitalized, 2 treated on intensive care unit) as well as group comparison at follow-up to matched healthy controls (12 participants, 7 male).*

| | **Post-COVID-19 subjects** | | | **Matched healthy controls** | |
|---|---|---|---|---|---|
| Quantity | Value at baseline | Value at follow-up | *p* value | Value | *p* value |
| | | | **Diffusion-weighted imaging** | | |
| Apparent diffusion coefficient (ADC, cm²/s, diffusion time 920 µs) | 0.0752 ± 0.0054 | 0.0773 ± 0.0057 | 0.38 | 0.0727 ± 0.0051 | 0.06 |
| ADC (cm²/s, diffusion time 1140 µs) | 0.0692 ± 0.0052 | 0.0713 ± 0.0053 | 0.21 | 0.0671 ± 0.0051 | 0.12 |

| | | | | | |
|---|---|---|---|---|---|
| ADC (cm²/s, diffusion time 1400 µs) | 0.0649 ± 0.0058 | 0.0664 ± 0.0045 | 0.16 | 0.0632 ± 0.0051 | 0.15 |
| ADC (cm²/s, diffusion time 1660 µs) | 0.0619 ± 0.0059 | 0.0631 ± 0.0051 | 0.21 | 0.0607 ± 0.0049 | 0.31 |
| ADC (cm²/s, diffusion time 1960 µs) | 0.0587 ± 0.0056 | 0.0602 ± 0.0053 | 0.15 | 0.0582 ± 0.0049 | 0.25 |
| Lung surface-volume ratio (1/cm) | 182.5 ± 23.5 | 173.0 ± 25.2 | 0.38 | 197.6 ± 20.0 | **0.02** |
| **Dynamic spectroscopy** | | | | | |
| Average RBC-TP ratio | 0.925 ± 0.196 | 0.999 ± 0.139 | **0.04** | 1.04 ± 0.17 | 0.54 |
| Relative RBC-TP oscillation amplitude (%) | 3.5 ± 0.9 | 3.6 ± 1.0 | 0.69 | 3.5 ± 0.5 | 0.62 |
| Absolute RBC-TP oscillation amplitude | 0.032 ± 0.010 | 0.035 ± 0.010 | **0.03** | 0.037 ± 0.007 | 0.58 |
| Average RBC-GP ratio | 0.145 ± 0.038 | 0.157 ± 0.037 | **0.01** | 0.141 ± 0.031 | 0.34 |
| Relative RBC-GP oscillation amplitude (%) | 4.2 ± 1.0 | 4.2 ± 1.0 | 0.73 | 4.1 ± 1.2 | 0.71 |

| | | | | | |
|---|---|---|---|---|---|
| Absolute RBC-GP oscillation amplitude | 0.0060 ± 0.0015 | 0.0065 ± 0.0019 | 0.11 | 0.57 ± 0.22 | 0.26 |
| Average TP-GP ratio | 0.160 ± 0.041 | 0.158 ± 0.032 | 0.95 | 0.135 ± 0.018 | 0.10 |
| Relative TP-GP oscillation amplitude (%) | 0.8 ± 0.2 | 0.6 ± 0.3 | 0.10 | 0.9 ± 0.4 | 0.07 |
| Absolute TP-GP oscillation amplitude | 0.0012 ± 0.0005 | 0.0009 ± 0.0005 | 0.23 | 0.0012 ± 0.0006 | 0.24 |
| FWHM RBC resonance (Hz) | 211.8 ± 10.0 | 207.0 ± 7.1 | 0.02 | 208.8 ± 9.8 | 0.69 |
| FWHM TP resonance (Hz) | 152.3 ± 9.4 | 154.7 ± 6.2 | 0.30 | 160.4 ± 7.2 | 0.34 |
| **Dissolved-phase imaging** | | | | | |
| Whole-lung RBC-TP | 0.254 ± 0.099 | 0.285 ± 0.060 | 0.12 | 0.302 ± 0.068 | 0.47 |
| Whole-lung RBC-GP (%) | 0.239 ± 0.094 | 0.279 ± 0.076 | **0.02** | 0.271 ± 0.074 | 0.89 |
| Whole-lung TP-GP (%) | 0.971 ± 0.230 | 0.976 ± 0.177 | 0.90 | 0.894 ± 0.140 | 0.21 |
| **Chemical shift saturation recovery** | | | | | |
| $\kappa$ (cm/s) | 0.061 ± 0.058 | 0.069 ± 0.061 | 0.76 | 0.051 ± 0.023 | 0.73 |
| $\eta$ | 0.280 ± 0.189 | 0.209 ± 0.063 | 0.21 | 0.247 ± 0.041 | 0.10 |
| $1/\tau$ (s) | 1.46 ± 0.77 | 2.22 ± 0.88 | **0.01** | 1.62 ± 0.48 | 0.06 |
| **Ventilation imaging** | | | | | |

| | | | | | |
|---|---|---|---|---|---|
| Ventilation defect percentage (%) | 22.5 ± 22.5 | 26.7 ± 19.0 | 0.21 | n/a | n/a |
| **Dynamic contrast-enhanced MRI** | | | | | |
| Mean pulmonary blood flow (ml/min/100ml) | 87.7 ± 26.9 | 87.5 ± 33.8 | 0.73 | n/a | n/a |
| **Cardiac MRI** | | | | | |
| Left ventricle | | | | | |
| Ejection fraction (%) | 54.2 ± 6.7 | 57.5 ± 5.7 | 0.20 | n/a | n/a |
| End-diastolic volume/body surface area (ml/m²) | 77.9 ± 11.4 | 78.4 ± 9.4 | 1.00 | n/a | n/a |
| End-systolic volume/body surface area (ml/m²) | 35.5 ± 6.5 | 33.2 ± 5.0 | 0.23 | n/a | n/a |
| Stroke volume/body surface area (ml/m²) | 42.4 ± 8.5 | 45.2 ± 7.8 | 0.47 | n/a | n/a |
| Myocardial mass/body surface area (g/m²) | 53.2 ± 8.3 | 52.8 ± 8.9 | 0.68 | n/a | n/a |
| Right ventricle | | | | | |

| | | | | | |
|---|---|---|---|---|---|
| Ejection fraction (%) | 55.5 ± 7.0 | 57.8 ± 5.6 | 0.52 | n/a | n/a |
| End-diastolic volume/body surface area (ml/m²) | 75.7 ± 13.0 | 79.3 ± 13.2 | 0.52 | n/a | n/a |
| End-systolic volume/body surface area (ml/m²) | 33.6 ± 8.2 | 33.6 ± 8.1 | 0.57 | n/a | n/a |
| Stroke volume/body surface area (ml/m²) | 42.1 ± 8.7 | 45.7 ± 7.9 | 0.32 | n/a | n/a |
| Myocardial mass/body surface area (g/m²) | 25.1 ± 4.3 | 24.3 ± 4.3 | 0.35 | n/a | n/a |

## References


1. Uecker M, Holme C, Blumenthal M, Wang X, Tan Z, Scholand N, et al. Mrirecon/BART [Internet]. 2021. DOI: 10.5281/zenodo.592960
2. Tsao J, Jiang Y. Hierarchical IDEAL: Fast, robust, and multiresolution separation of multiple chemical species from multiple echo times. Magn Reson Med. 2013;70:155–9. DOI: 10.1002/mrm.24441
3. Sotak CH, Dumoulin CL, Newsham MD. Automatic phase correction of fourier transform NMR spectra based on the dispersion versus absorption (DISPA) lineshape analysis. J Magn Reson. 1984;57:453–62. DOI: 10.1016/0022-2364(84)90260-9

4. Vogel-Claussen J, Finn JP, Gomes AS, Hundley GW, Jerosch-Herold M, Pearson G, et al. Left Ventricular Papillary Muscle Mass. J Comput Assist Tomogr. 2006;30:426–32. DOI: 10.1097/00004728-200605000-00013
